# The Role of Diacylglycerol Acyltransferase (DGAT) 1 and 2 in Cardiac Metabolism and Function

**DOI:** 10.1038/s41598-018-23223-7

**Published:** 2018-03-21

**Authors:** Nathan D. Roe, Michal K. Handzlik, Tao Li, Rong Tian

**Affiliations:** Mitochondria and Metabolism Center, Department of Anesthesiology and Pain Medicine, University of Washington, Seattle, WA 98109 USA

## Abstract

It is increasingly recognized that synthesis and turnover of cardiac triglyceride (TG) play a pivotal role in the regulation of lipid metabolism and function of the heart. The last step in TG synthesis is catalyzed by diacylglycerol:acyltransferase (DGAT) which esterifies the diacylglycerol with a fatty acid. Mammalian heart has two DGAT isoforms, DGAT1 and DGAT2, yet their roles in cardiac metabolism and function remain poorly defined. Here, we show that inactivation of DGAT1 or DGAT2 in adult mouse heart results in a moderate suppression of TG synthesis and turnover. Partial inhibition of DGAT activity increases cardiac fatty acid oxidation without affecting PPARα signaling, myocardial energetics or contractile function. Moreover, coinhibition of DGAT1/2 in the heart abrogates TG turnover and protects the heart against high fat diet-induced lipid accumulation with no adverse effects on basal or dobutamine-stimulated cardiac function. Thus, the two DGAT isoforms in the heart have partially redundant function, and pharmacological inhibition of one DGAT isoform is well tolerated in adult hearts.

## Introduction

Cardiac triglyceride (TG) accumulation is a common clinical feature observed in older individuals and patients with diabetes^1,2^. Although increased cardiac TG content has previously been considered as a marker of lipotoxic cardiomyopathy, recent work showed that enhancing cardiac triglyceride production by diacylglycerol:acyltransferase 1 (DGAT1) overexpression was protective against lipotoxicity and ischemia-reperfusion injury^3,4^. These findings suggest that cardiac lipid turnover plays an important role in stress response. When cardiac TG lipolysis is blocked by deletion of adipose triglyceride lipase (ATGL) it results in a dramatic steatosis, cardiac dysfunction and early death^5^. Alternatively, increasing cardiac lipolysis by overexpression of ATGL protects against pressure overload and diabetic cardiomyopathy^6,7^. These data suggest that TG synthesis followed by breakdown, otherwise known as TG turnover, plays a pivotal role in cardiac physiology and diseases. Although studies targeting TG metabolism through ATGL or DGAT1 overexpression have been enlightening, few studies exist that have evaluated the effect of decreased TG production on cardiac metabolism and health. This is an important issue as inhibition of TG synthesis is being considered as a therapeutic measure for hyperlipidemia^8–10^.

The final step in TG synthesis is catalyzed by DGAT 1 and 2, both of which are present in the heart. The DGAT1-null mice were previously shown to be resistant to diet-induced obesity with no cardiac phenotype, mainly due to reduced lipid uptake in the intestine^9–12^. The DGAT1-null mice had normal levels of TG and diacylglycerol (DAG) in the heart^10^ while cardiac specific deletion of DGAT1 was sufficient to increase DAG levels without effecting TG levels^13^. The *in vivo* rate of TG synthesis or turnover in the heart has never been assessed in these models. The relative contribution of the two DGAT isoforms in cardiac triglyceride metabolism has not been addressed either.

In addition to protection against lipotoxicity, TG turnover has been suggested to be critical for modulation of fatty acid oxidation via regulating the transcriptional activity of peroxisome proliferator-activated receptor α (PPARα), possibly by contributing a fatty acid ligand. Previous studies have shown that either extracellular TG lipolysis by lipoprotein lipase (LPL) or intracellular TG lipolysis by ATGL can increase PPARα activity^5,14,15^. In rodent hearts, TG turnover rate was shown closely correlated with PPARα activity and cardiac function^5,14,15^. In failing human hearts, DGAT expression was shown to be decreased which was considered a pathogenic mechanism for decreased fatty acid oxidation and lipotoxicity^16^.

In the present study we determined the role of DGAT 1 and 2 in TG synthesis and turnover in the heart as well as their contributions to cardiac fatty acid metabolism. Utilizing an inducible cardiac-specific DGAT1 deletion mouse model (iKO) together with DGAT2-specific inhibitor, we were able to achieve graded inhibition of TG synthesis and turnover in adult mouse hearts. We observed that deletion of DGAT1 only modestly reduced TG synthesis from exogenous fatty acids which correlated with increased fatty acid oxidation and had no effect on cardiac function up to 1 year of age. Furthermore, we found that DGAT2 compensated for DGAT1 function in iKO and that inhibition of both isoforms abrogated TG synthesis but did not alter cardiac response to high fat diet.

## Results

### Deletion of DGAT1 has no effect on cardiac TG content and function

**Figure 1:**
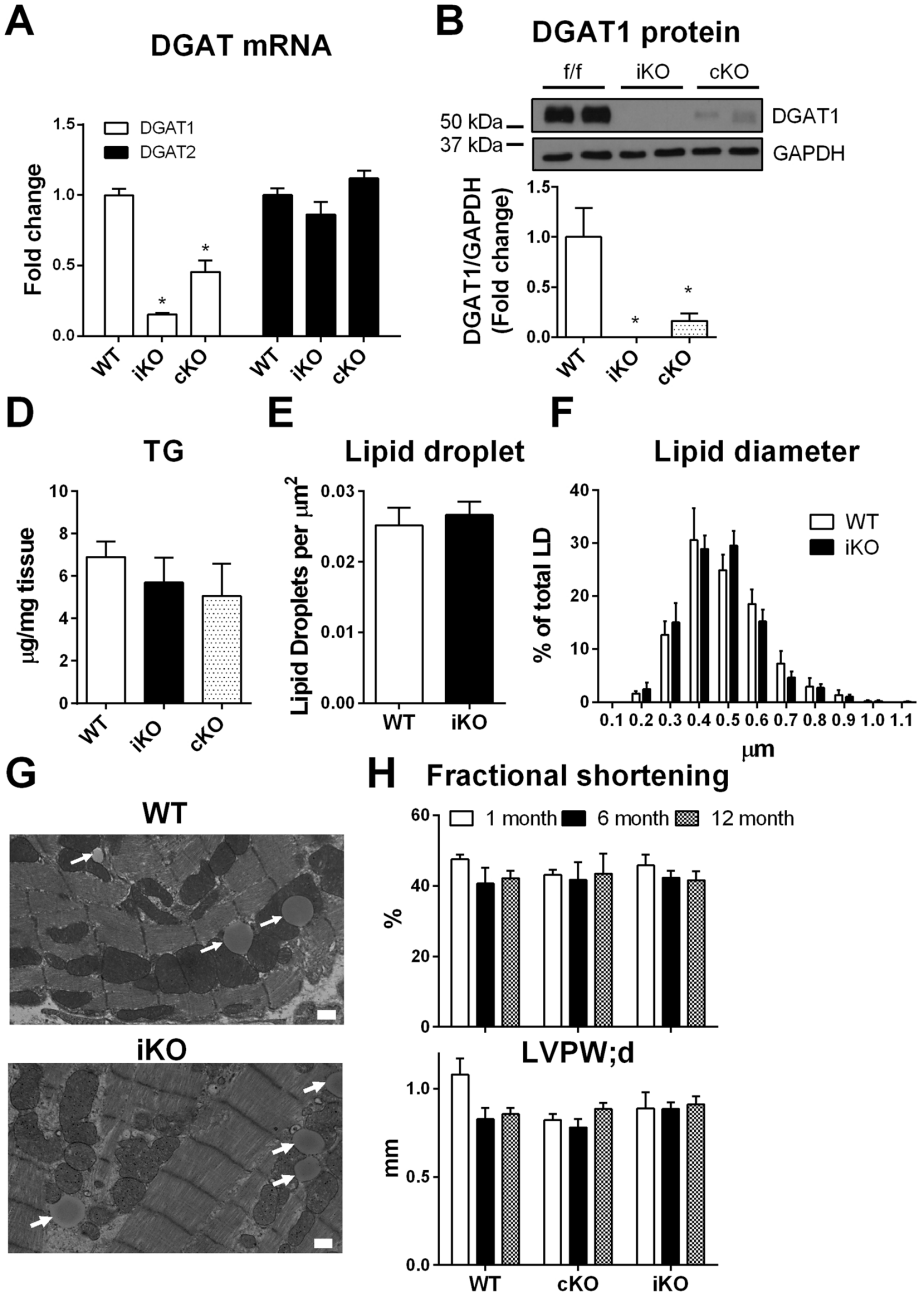
Triglyceride storage is unaffected in DGAT1 inducible knockout (iKO) mice. Effective knockdown of DGAT1 in the heart was achieved as evidenced by significant reduction in DGAT1 mRNA (**A**) and protein (**B**), which was not accompanied by a concomitant increase in DGAT2 mRNA (**A**). Although DGAT1 mRNA was reduced, there was no change in cardiac triglyceride content in iKO hearts compared to control (**D**). Electron microscopic analysis also revealed the presence of lipid droplet in iKO hearts at comparable sizes to control (**E**–**G**). Fractional shortening and left ventricular posterior wall thickness demonstrated unaltered cardiac function in cardiac-specific constitutive (cKO) and iKO mice (**H**). Full-length blots are presented in Supplementary Figure 4. Data are presented as mean ± SEM (n = 3–11). *P < 0.05 vs. control.

To investigate the role of DGAT1 in the heart, we generated cardiac-specific constitutive and inducible DGAT1 KO mouse models (cKO and iKO, respectively). Both models showed reduced DGAT1 mRNA (Fig. 1A) and protein (Fig. 1B) with no effects on DGAT2 mRNA expression or cardiac TG content (Fig. 1A,D). As evidenced by electron microscopy, there were no differences between genotypes for cardiac lipid droplet number and morphology (Fig. 1E–G). Relative to control mice, echocardiography analysis showed no differences in cardiac geometry or function of cKO and iKO mice for up to 12 months (Fig. 1H, Supplemental Table 2). To focus on the effects of DGAT inhibition in adult hearts, we used iKO model for the rest of the study.

### Cardiac TG synthesis is modestly reduced with loss of DGAT1

**Figure 2:**
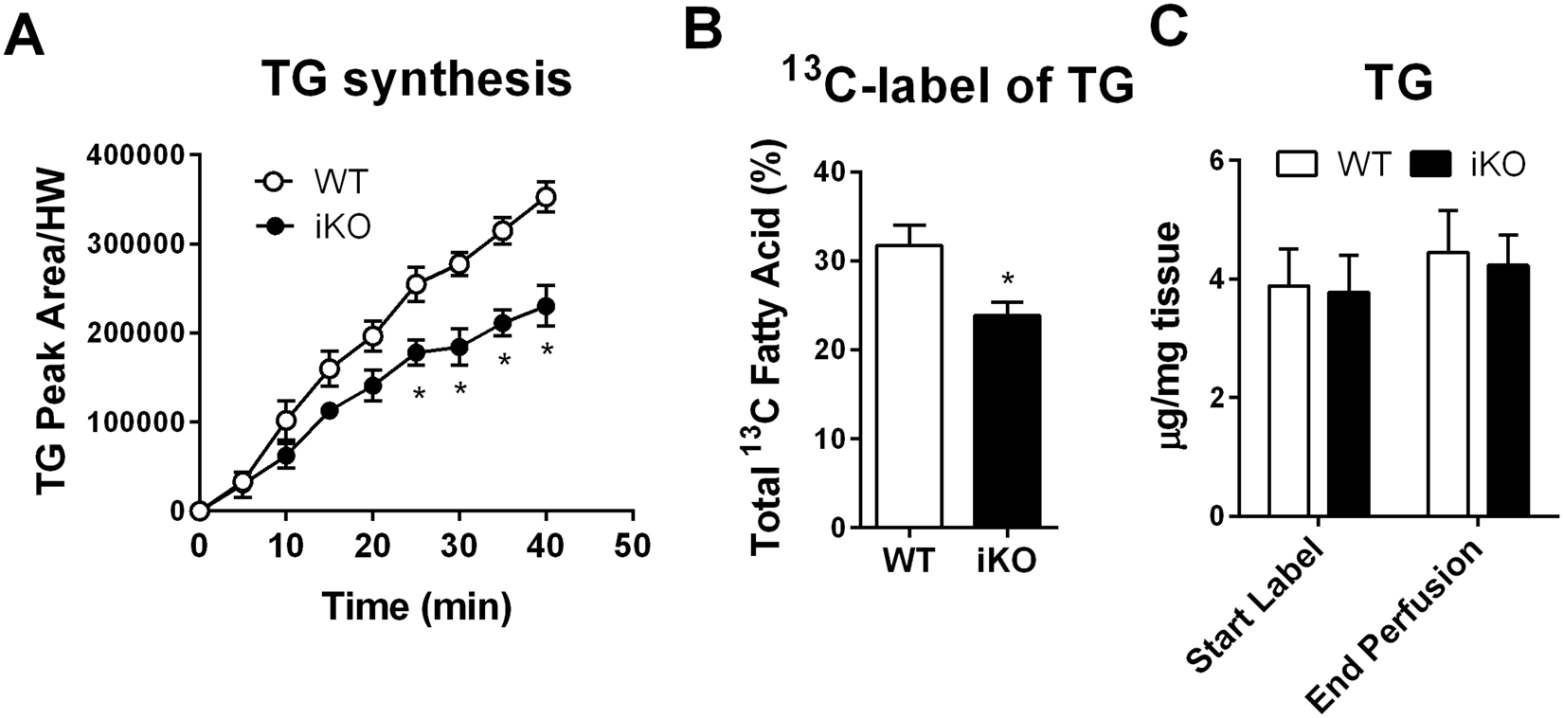
TG formation in the perfused heart is significantly reduced in inducible DGAT1 knockout animals. ^13^C labeling of the triglyceride pool in perfused hearts from iKO animals revealed that loss of DGAT1 reduces TG synthesis rate (**A**). GCMS analysis of the triglyceride pool confirmed only a modest reduction of ^13^C labeling in iKO (**B**). This was observed while TG levels remained constant during the perfusion period (**C**). Data are presented as mean ± SEM (n = 3–9). *P < 0.05 vs. control at a given time point.

Using ^13^C NMR spectroscopy we examined fatty acid incorporation rate into the TG pool of isolated hearts perfused with ^13^C labeled substrates. The iKO showed a 30% lower rate of TG synthesis from exogenous fatty acids than the control (Fig. 2A). GC-MS analysis of cardiac triglycerides extracted from the heart at the end of labeling confirmed reduction in ^13^C labeled fatty acids in the TG pool (Fig. 2B). Moreover, total cardiac TG content was unaffected by inducible DGAT1 deletion (Fig. 2C). Collectively, these data show a modest decrease in cardiac TG turnover following DGAT1 deletion in adult hearts.

### Increased oxidation of exogenous fatty acids in DGAT1 iKO hearts

**Figure 3:**
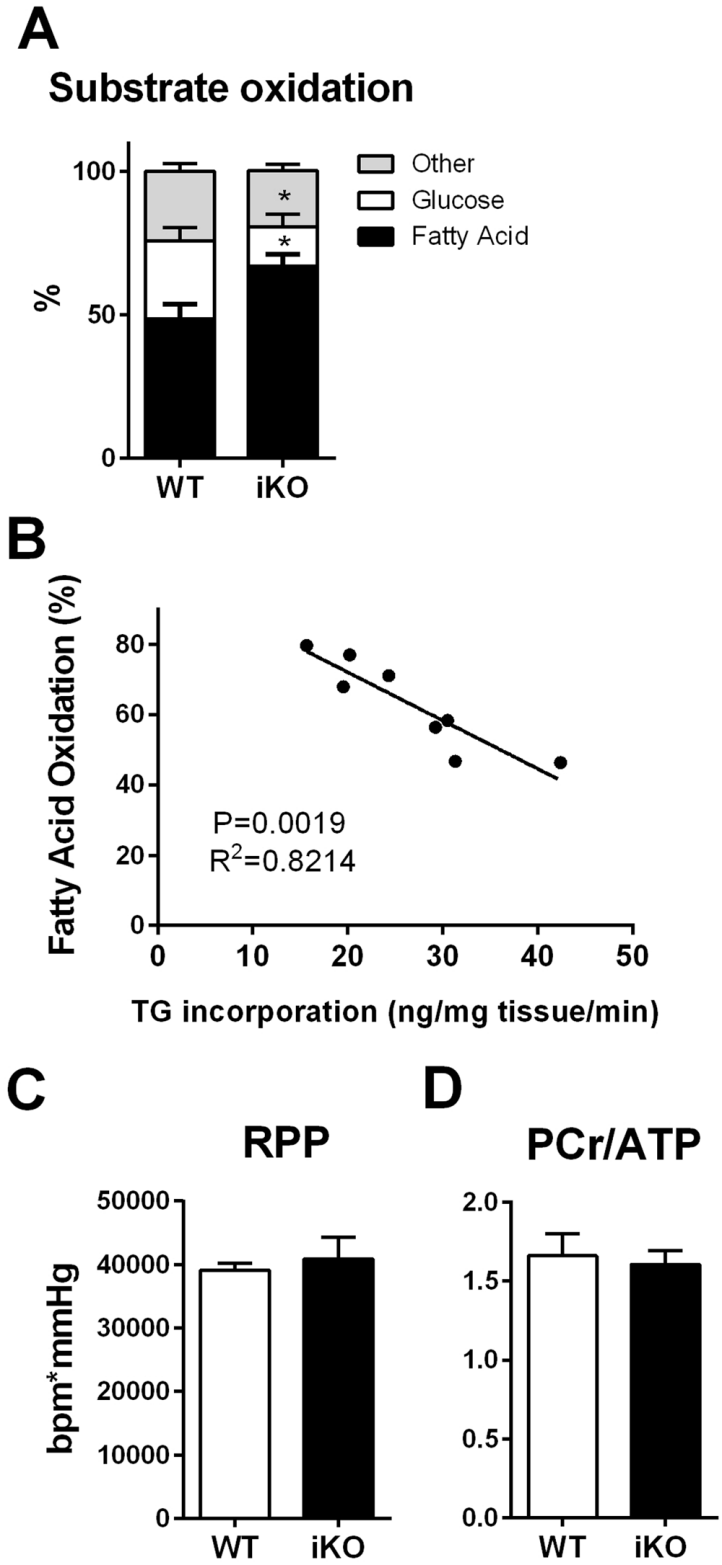
Inducible DGAT knockout alters cardiac metabolism and reduces triglyceride fatty acid incorporation. ^13^C isotopomer analysis of glutamate revealed increased fatty acid and decreased glucose oxidation in iKO hearts compared to control (**A**). A Pearson’s correlation coefficient shows a strong correlation between TG incorporation and fatty acid oxidation (**B**), suggesting these two pathways are interconnected in governing the overall fate of fatty acid in the heart. No alteration in cardiac function (**C**) or energetics (**D**) was observed in the perfused hearts in between genotypes. Data are presented as mean ± SEM (n = 4–8). In (A) *P < 0.05 vs. control.

To assess the effects of reduced TG turnover on substrate oxidation, the relative contribution of ^13^C labeled fatty acids and glucose along with unlabeled substrates to the tricarboxylic acid (TCA) cycle was assessed by ^13^C-NMR techniques. Compared to control, increased contribution from exogenous fatty acid and reduced glucose contribution to total oxidative metabolism was observed in iKO hearts (Fig. 3A). Moreover, there was a negative correlation between fatty acid oxidation and the incorporation of exogenous fatty acids into the TG pool (Fig. 3B), suggesting that decreased TG synthesis is associated with increased oxidation of exogenous fatty acids. These changes in fatty acid metabolism did not affect cardiac function or energetics in the perfused iKO hearts (Fig. 3C,D). Additionally, these hearts responded normally to increased workload *in vivo* induced by dobutamine (Fig. S1). These data suggest that adult hearts are able to adapt to a moderate decrease in TG turnover caused by DGAT1 deletion and are able to maintain cardiac energetics or function.

### DGAT1 iKO hearts respond normally to high fat diet

**Figure 4:**
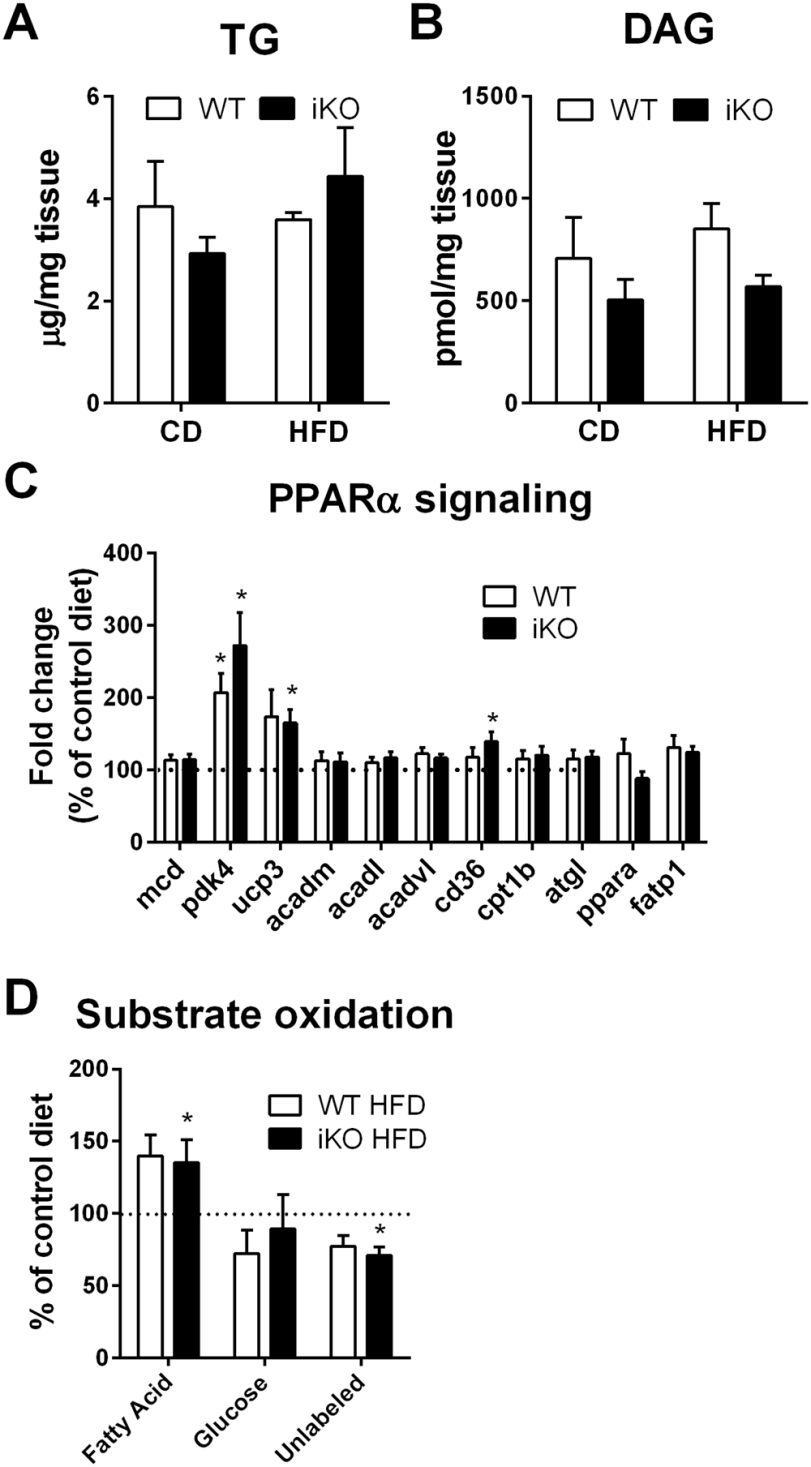
DGAT1 deficiency shows no effect on cardiac lipid metabolism after a short term high fat diet (HFD) feeding. Cardiac TG (**A**) and DAG (**D**) contents were not different between control and iKO mice following HFD. PPARα target gene expression (**C**) and fatty acid contribution to total oxidative metabolism (**D**) were not different between WT and iKO mice in response to HFD. The dotted line indicates the level of chow-fed controls. Data are presented as mean ± SEM (n = 6–10). *P < 0.05 vs. control - Control Diet.

To evaluate the effect of reduced TG synthesis on lipid loading-induced metabolic remodeling, we subjected control and iKO mice to a high fat diet (HFD, 60% kcal from fat) for 5 weeks. Body weight gains were similar in control and iKO mice after the 5 week HFD (Fig. S2A-B). Cardiac TG or DAG content did not change in control or iKO hearts after 5 weeks of a high fat diet (Fig. 4A,B). However, the 5-week HFD was sufficient to stimulate PPARα activity and increase fatty acid oxidation as previously described^17^. Interestingly, increases in PPARα activity and fatty acid oxidation were similar in iKO and control hearts (Fig. 4C,D) suggesting that the modest reduction in triglyceride turnover did not affect hearts’ ability to activate PPARα and increase fatty acid oxidation in response to increased lipid load.

### Role of DGAT2 in cardiac TG metabolism

**Figure 5:**
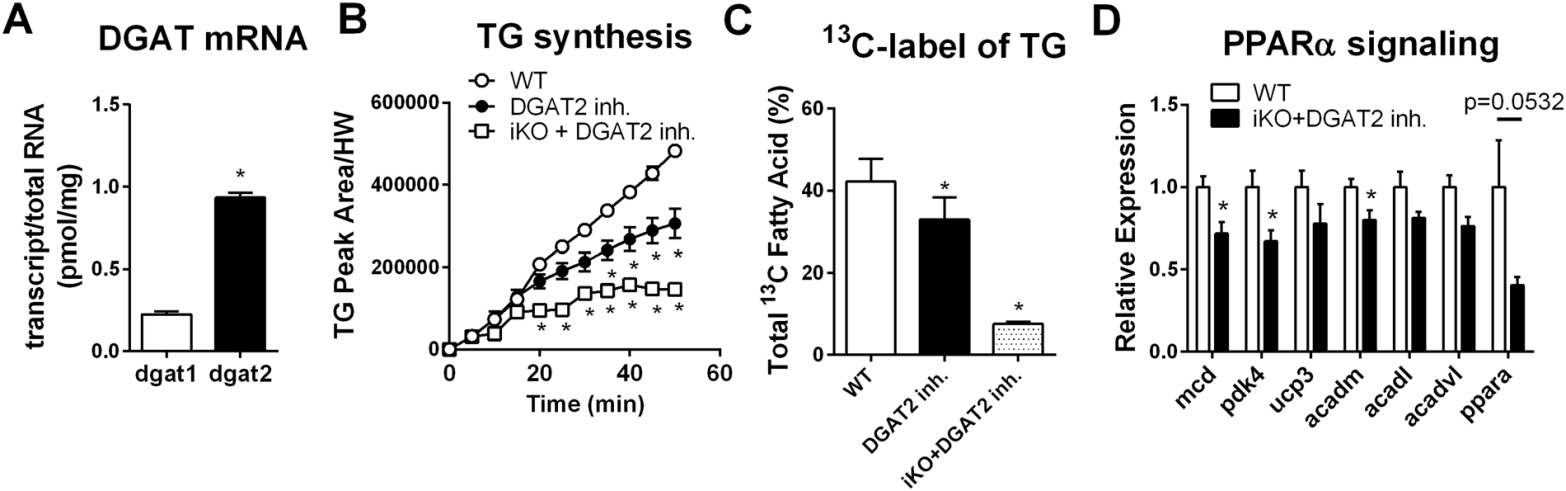
Contribution of individual DGAT isoforms to triglyceride metabolism. mRNA quantification of both DGAT isoforms in the mouse heart (**A**), ^13^C labeling of TG pool in the control and iKO hearts perfused with DGAT2 inhibitor (**B**), mass spectrometry analysis of ^13^C labeling of TG pool in control and iKO hearts perfused with DGAT2 inhibitor (**C**) and PPARα signaling between WT and iKO hearts perfused with 5 µM DGAT2 inhibitor (**D**). Data are presented as mean ± SEM (n = 3–7). *P < 0.05 vs. control

Considering relatively mild effects of DGAT1 deficiency on cardiac lipid metabolism, we next sought to explore the relative contribution of the two DGAT isoforms in the heart. Quantitative measurements of the mRNA levels showed greater abundance of DGAT2 mRNA in the heart compared to DGAT1 (~1 vs. ~0.25 pmol/mg RNA respectively, Fig. 5A). To evaluate the contribution of DGAT2 to TG synthesis, we perfused hearts from control or iKO mice with DGAT2 inhibitor (5 µM)^18^. The DGAT2 inhibitor alone has very modest effect but inhibition of both isoforms substantially reduced ^13^C fatty acid incorporation into TG pool in the heart as measured by NMR spectroscopy and GCMS (Fig. 5B,C). Moreover, perfusion with DGAT2 inhibitor for 60 minutes did not alter cardiac TG levels in control or iKO hearts (Fig. S3A) suggesting that inhibition of both DGAT isoforms effectively abrogated TG turnover in the heart. Furthermore, we found that inhibition of TG turnover resulted in a rapid downregulation of PPARα target gene expression with no change in cardiac function (Fig. 5D and Fig. S3B), and DGAT2 inhibitor did not further change substrate oxidation in iKO during the perfusion period (Fig. S3C).

### Cardiac effects of DGAT1/2 inhibition *in vivo*

**Figure 6:**
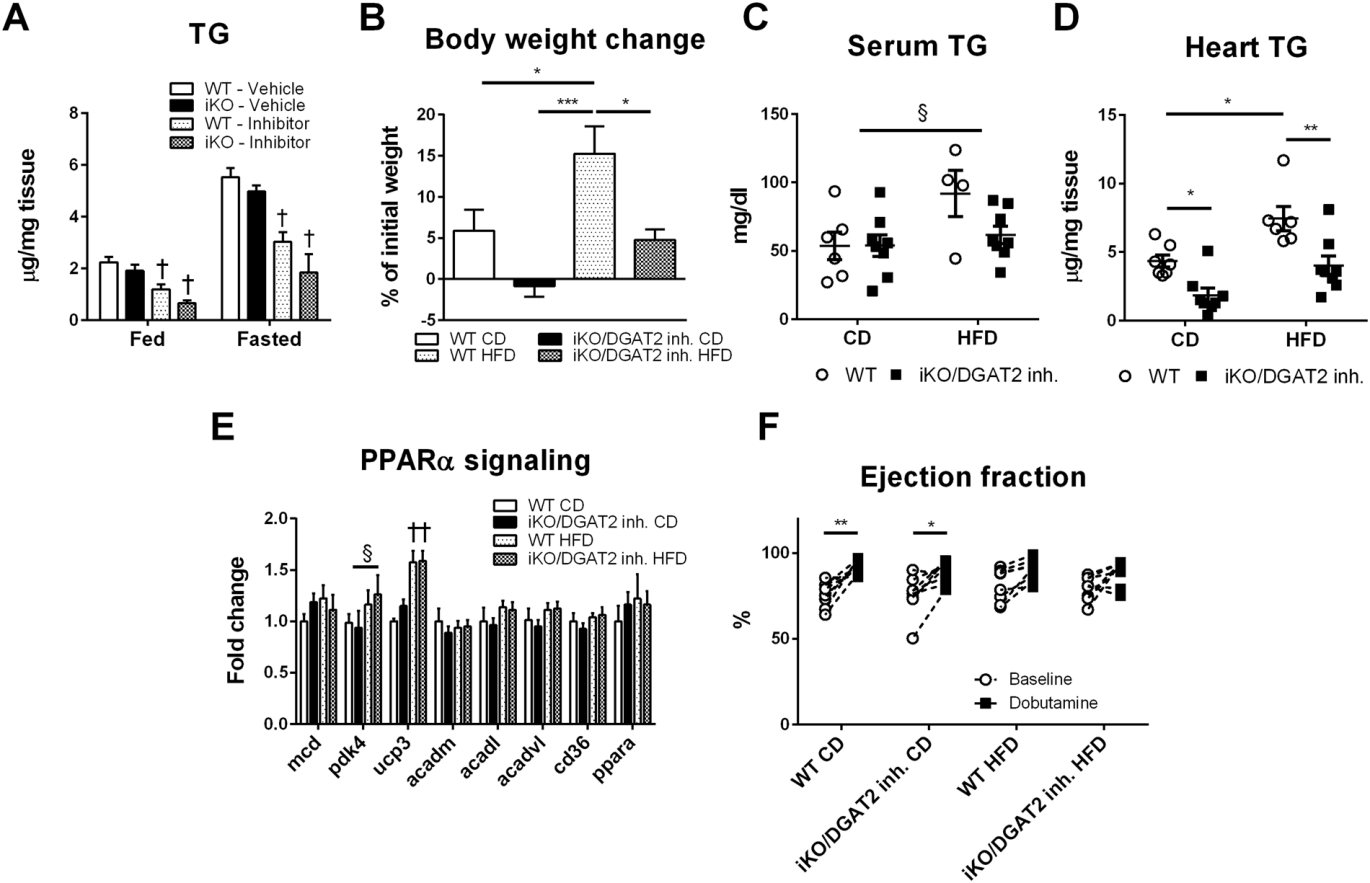
Coinhibition of DGAT1 and DGAT2 during high fat diet (HFD). Cardiac TG level was decreased with DGAT2 inhibition or with iKO + DGAT2 inhibition under fed and fasting conditions (**A**). Coinhibition of DGAT1 and DGAT2 attenuated body weight gain (**B**) and reduced serum (**C**) and cardiac (**D**) TG content following HFD. PPARα signaling (**E**) and basal cardiac function (**F**) were unaffected in iKO + DGAT2 mice on a HFD compared to control. Dobutamine-induced increase in cardiac function was attenuated by HFD with no differences between genotypes (**F**). Data are presented as mean ± SEM (n = 4–8). *P < 0.05, **P < 0.01, ***P < 0.001. ^†^P < 0.05 vs. corresponding control group, ^§^P < 0.05 mean effect of diet. Data analyzed by one-way ANOVA (A) and two-way ANOVA (**B**–**F**).

To explore how DGAT1/2 coinhibition modulates *in vivo* cardiac lipid metabolism, we treated iKO mice with DGAT2 inhibitor for 5 days. Under fasting and fed conditions, both control and iKO mice treated with DGAT2 inhibitor had lower cardiac triglyceride content compared to vehicle treated controls (Fig. 6A). To investigate whether prolonged DGAT1/2 coinhibition modifies cardiac lipid metabolism and function during sustained lipid overload we subjected mice to 5-week HFD. Treatment with DGAT2 inhibitor in mice on control diet showed a modest effect on body weight with no significant change of blood TG level. In HFD-fed mice, however, DGAT2 inhibitor reduced body weight gain and protected against the increase of blood TG level (Fig. 6B,C). Cardiac TG was also lower in iKO mice treated with DGAT2 inhibitor (Fig. 6D). The downregulation of cardiac TG level did not affect cardiac PPARα signaling or its response to HFD (Fig. 6E). Interestingly, while basal cardiac function was unaltered between genotypes on control diet or HFD, dobutamine-induced increase in ejection fraction was attenuated in mice subjected to the HFD which was not altered by DGAT inhibition (Fig. 6F).

## Discussion

In this study we show that both isoforms of DGAT contribute to triglyceride synthesis in the heart with partially redundant function. Loss of DGAT1 in the adult mouse heart exerts a moderate inhibition on cardiac triglyceride synthesis accompanied by increased oxidation of exogenous fatty acids. Inhibition of both enzymes abrogates triglyceride synthesis and turnover in the perfused heart leading to the downregulation of PPARα target gene expression. Treatment with DGAT2 inhibitor in mice with cardiac-specific DGAT1 deficiency prevents TG accumulation in the heart during 5-week high fat diet. It however, does not change the PPARα signaling or cardiac performance under control or high fat diet conditions.

It has been well recognized that myocardial TG pool is highly dynamic although the heart has very limited capacity for lipogenesis. The exact role of the TG turnover is not entirely clear but several potential functions have been proposed including preventing the generation of lipotoxic species during lipid overload, and supply fuel for oxidation upon energy stress. Several recent studies have suggested that TG lipolysis may regulate fatty acid oxidation by supplying ligands for PPARα activation^5^. Critical evidence in support of this notion was derived from mice with ATGL deletion in which blockade of TG lipolysis resulted in decreased fatty acid oxidation that could be rescued by pharmacological activation of PPARα. However, blockade of TG lipolysis also causes substantial steatosis that confounds the ATGL deletion phenotype. In this study, we find that TG turnover can be inhibited by targeting TG synthesis with no change of TG pool size. Using this approach, we show that partial inhibition of TG turnover by deleting DGAT1 in adult mouse heart does not impair PPARα transcriptional activity. The heart adapts to the decreased TG synthesis by increasing oxidation of the exogenous fatty acids without changes in lipid metabolism homeostasis or myocardial energetics. In response to high fat diet, not only does DGAT1 deficiency not affect cardiac function, but the iKO heart retains its ability to upregulate PPARα activity and remodel metabolism similar to that of the WT. This is unexpected given the growing body of evidence suggesting a close association of TG metabolism and PPARα activity with either TG turnover regulating PPARα activity^5,15,19^, or PPARα activity itself increasing TG turnover^20^. Furthermore, contrary to the previous studies suggesting that reduced TG synthesis and turnover would increase lipotoxic species in the heart^3,13,21^, DAG content in the iKO hearts did not change at baseline or following a high fat diet. These observations have led us to conclude that inhibition of DGAT1 in the adult heart exert insufficient effect on TG synthesis rates to impair cardiac function, lipid homeostasis, or PPARα activation.

Previous studies have revealed important differences in structure and subcellular localization of the two DGAT enzymes, suggesting that DGAT1 and 2 participate in different pathways of TG synthesis^22,23^. A model based on this premise proposes that DGAT1 is primarily involved in esterifying exogenous fatty acids to replenish the TG pool while DGAT2 is responsible for incorporating endogenously synthesized fatty acids into TG^22^. Since the heart is not a lipogenic organ, DGAT1 would be expected to play a primary role in TG synthesis. Interestingly, DGAT2 is highly expressed in the heart but its function has not been assessed due to neonatal lethality of DGAT2 knockout mice^24^. Using dynamic ^13^C-NMR technique, here we show that the two enzymes have partially redundant function in the heart; inhibition of either enzyme only modestly affects the rate of fatty acids incorporation into the TG pool. Inhibition of both DGAT activities results in near complete loss of TG synthesis in the heart. This is consistent with the profound reduction of TG in fetal mice lacking both enzymes^25^, thus, supporting the notion that these two DGATs are jointly responsible for TG synthesis in the heart. Compensation by DGAT2 is likely an adaptive mechanism for DGAT1 deficiency in the heart. Although deletion of DGAT1 in the adult mouse hearts does not trigger any increases of DGAT2 mRNA level, we are unable to determine the protein level or post-translational modification of DGAT2 in the iKO hearts due to the lack of DGAT2-specific antibody. Recent studies have shown that ubiqutination or translocation of DGAT2 could change its stability or function in non-cardiac cells^26^, whether a similar mechanism contributes to cardiac triglyceride synthesis warrants further investigation.

Targeting TG synthesis has been proposed as a therapeutic strategy for hyperlipidemia for more than a decade^9^. Multiple small molecular inhibitors of DGAT, especially DGAT2, have been developed recently^18,27,28^. However, previous studies have suggested that inhibition of TG synthesis and turnover in the heart could contribute to lipotoxicity and hence cautioned the use of pharmacological inhibition of DGAT. In contrast, we find that inhibition of one isoform of DGAT in the adult heart is well tolerated. Once a near complete inhibition of TG synthesis and turnover is achieved by perfusing iKO hearts with DGAT2 inhibitor, expression of PPARα target genes is significantly downregulated. Inhibition of both DGAT isoforms *in vivo*, although effectively reduces TG content in the heart, do not affect PPARα activity or cardiac function under normal or high fat diet condition. We speculate that pharmacological inhibition of DGAT2 in the heart is only intermittent *in vivo*, thus resulting in a state of partial suppression of TG turnover despite the reduction of TG level. Collectively, these observations suggest that a low rate of TG turnover is sufficient to maintain PPARα activity, and furthermore, adult hearts can adapt to partial inhibition of DGAT activity. It remains to be determined whether this degree of DGAT inhibition affects cardiac responses to other chronic stresses such as pressure overload.

## Conclusions

In the present study, we find that both isoforms of DGAT contribute to cardiac TG synthesis with partial redundancy. Coinhibition of DGAT1 and 2 in the heart is necessary to abrogate cardiac TG turnover. Moreover, inhibiting DGAT1 and 2 in the heart during chronic, sustained lipid loaded protects against cardiac lipid accumulation without significant changes of cardiac function.

## Methods

### Animal models

All animal procedures were approved by the University of Washington Institutional Animal Care and Use Committee (IACUC) and were performed in accordance with IACUC guidelines and regulations. DGAT1 flox/flox mice were purchased from Jackson Labs and crossed with either α-MHC-MerCreMer (MCM) transgenic mice to generate cardiac-restricted inducible DGAT1 knockout (iKO) or with α-MHC-CRE transgenic mice to generate cardiac-specific DGAT1 knockout (cKO). Age and sex-matched littermate DGAT1 flox/flox mice were used as controls. For iKO mice, tamoxifen (20 mg/kg/d, i.p.) was given for 5 days to 8-week old MCM-DGAT1 flox/flox mice and mice were analyzed 4 weeks after final tamoxifen injection. For high fat diet treatment, 3-month old mice were fed a diet with 60.3% kcal from fat (HFD, Research Diets TD.09766) or a control diet with 16.8% kcal from fat (CD, Research Diets TD.120455) for 5 weeks. For DGAT2 inhibitor treatment, control or iKO mice 4 weeks after tamoxifen treatment were orally gavaged (30 mg/kg, BID) of the DGAT2 inhibitor PF-06424439-27 in 0.5% methylcellulose for 5 days and tissues harvested with or without overnight fasting. Separate cohort of mice on HFD or CD were treated with the same dose of PF-06424439-27 for 5 weeks. All mice were kept on a 12 h light/dark cycle with *ad libitum* access to food and water.

### Echocardiography

Transthoracic echocardiography was collected from mice anesthetized with isoflurane (1–2%) using a Vevo 2100 (VisualSonics) in short axis mode. Dobutamine stress echo was conducted by administration of dobutamine (3 mg/kg, i.p.) immediately after the baseline function assessment and repeating the echocardiographic measurements 10 minutes after dobutamine injection. Data for each mouse is an average of 3 cardiac cycles taken at comparable heart rates.

### Perfusion and Substrate Utilization

Langendorf perfusion was carried out as previously described^4,29,30^. Mice were heparinized (100 U/mouse), anesthetized with pentobarbital (190 mg/kg, I.P.), hearts excised and perfused at 80 mmHg perfusion pressure at 37 °C with a KH buffer containing the following in mM: 0.5 EDTA, 5.3 KCl, 1.2 MgSO4, 118 NaCl, 25 NaHCO3, 2 CaCl2, 0.4 mixed long chain fatty acids, 5.5 glucose, 1.2 lactate and 50 µU/mL insulin. *Ex vivo* cardiac phosphocreatine (PCr) and ATP contents were determined using ^31^P NMR as previously described^4^. Contribution of individual substrates to total oxidative metabolism was determined using ^13^C NMR^4^. Specifically, hearts perfused with 1,6-^13^C glucose and U-^13^C mixed fatty acids were freeze-clamped following 40 min labelling perfusion, tissues were powdered and metabolites extracted with perchloric acid. Proton-decoupled ^13^C NMR spectra of tissue extracts were acquired on a 14 T magnet using TopSpin software (Bruker 600; Billerica, MA) and contributions of labeled (glucose and fatty acids) and unlabeled (exogenous lactate and endogenous glycogen and TG) substrates to total oxidative metabolism were determined by modeling of citric acid cycle flux using the glutamate C3 and C4 isotopomer peak areas (tcaCALC, Dallas, TX).

### TG synthesis

Incorporation of ^13^C-labeled mixed fatty acids into the TAG pool was analyzed by monitoring the resonance of the methylene carbon at ~31 ppm via ^13^C NMR spectroscopy as previously described^4^. Proton-decoupled carbon spectra were determined on isolated hearts perfused with ^13^C-labeled mixed fatty acids over a 60-min period. Peak areas of the methylene resonance of each sequential spectrum were quantified using software from Advanced Chemistry Development Laboratories (ACD Laboratories, Toronto, Ontario, Canada) and corrected for ^13^C natural abundance.

### Electron Microscopic Analysis

Thin heart sections were cut and rinsed in a KH buffer containing 20 mM KCl to arrest the muscle in diastole. Sections were immediately placed into a fixation solution containing 0.4% glutaraldehyde in 0.1 M sodium cacodylate buffer. Fixed tissue samples were processed and images taken using a Transmission Electron Microscope (JEOL1230). Lipid droplet number and morphology were quantified using ImageJ analysis software. Data for each heart were calculated as average value of 40 random images.

### Biochemical Assays

Total lipids were extracted using the Folch extraction procedure and quantified using the sigma colorimetric assay kit as previously described^31^. Briefly, heart tissues were weighed and homogenized in 2:1 chloroform:methanol, incubated on ice for 30 min, then centrifuged at 3000 rpm for 12 minutes. The top layer was discarded, the bottom layer was dried under N2 gas and reconstituted in 1% Triton X-100:Isopropanol. Individual lipid classes were extracted as described above were loaded onto an NH2 column (Agilent BondElut) preconditioned with hexane and neutral lipids were extracted with 2:1 Chlorofom:Isopropanol, dried under N2 gas, reconstituted in hexane and loaded onto another preconditioned NH2 column^4,32^. TG were eluted from the column by the addition of 1% diethyl ether:10% methylene chloride in hexane and DAG with 15% ethyl acetate in hexane dried under N2 gas. DAG was quantified using the triglyceride assay kit with a DAG standard curve. Fatty acid composition and ^13^C labeling patterns was assessed using a gas chromatograph mass spectrometer (GCMS, Shimadzu QP2010) with a 0.25 mm inner diameter column (Restek Rtx-225) on fatty acid methyl esthers (FAMES) made from the triglyceride fraction obtained using solid phase separation as previously described^33^.

### Real Time PCR

RNA from frozen heart tissue was isolated using the RNeasy fibrous extraction kit (Qiagen) and cDNA was synthesized using the Omniscript (Qiagen) reverse transcriptase according to the manufacturer’s recommendations. PCR reactions containing equal cDNA concentrations were set up in duplicate using Sybr green (BioRad) on a rotogene using concentrated cDNA as a standard. All values were normalized to 18 S concentration and expressed as a fold change from control. Primer sequences for all genes are described in Supplemental Table 1. Quantitative measurements of DGAT1 and DGAT2 levels in mouse heart tissue were performed based on standard curves generated using full-length amplicons supplied by Integrated DNA technologies.

### Western blotting

Whole heart proteins were extracted from flash frozen hearts using 10–20 mg of wet weight tissue using RIPA buffer containing 150 mM NaCl, 50 mM Tris-HCl, 1% Triton X-100, 0.1% SDS, 0.1% sodium deoxycholate and 1 mM EDTA, pH 7.4. Briefly, frozen hearts were homogenized in RIPA buffer using bullet blender and placed on ice for 30 min. Next, samples were centrifuged for 10 min at 4 °C at 20,000 g. Supernatant was collected and its protein content determined using BCA assay kit (BCA protein assay kit, Pierce Biotechnology, USA). Protein samples were run a 10% Bis-Tris acrylamide gel for 2 h at constant 100 V and transferred on a polyvinylidenedifluoride membrane (PVDF) for 2 h at constant 250 mA in an ice-chilled transfer tank. The membrane was blocked and incubated overnight at 4 °C with mouse anti-DGAT1 antibody (Santa Cruz, #sc-271934) and rabbit anti-GAPDH antibody (Cell Signaling, #D16H11). Membranes were then washed and incubated with corresponding anti-mouse or goat anti-rabbit HRP-conjugated secondary antibody (R&D Systems). After washing membranes were incubated with enhanced chemiluminesence (ECL) detection solution (Supersignal Westpico Chemiluminescent Substrate, Thermo, #34080) and exposed to X-ray film (HyBlot CL, Denville Scientific, Inc., NJ, US). Intensity was measured by ImageJ densitometry software.

### Statistics

All data are presented as mean ± SEM. Statistical analysis was conducted using a student’s t-test for experiments with 2 groups, one-way ANOVA was used for experiments of three or more groups. Two-way ANOVA was used for experiments with repeated measures as specified in the figure legend. Graphs and statistical analyses were performed using Graph Pad Prism 6.0. Statistical significance was set at P < 0.05.

### Data availability

All data generated or analyzed during this study are included in this published article (and its Supplementary Information files).

## Electronic supplementary material

Supplementary Dataset
