# Supplementary material for: The Role of Diacylglycerol Acyltransferase (DGAT) 1 and 2 in Cardiac Metabolism and Function
Source: Sci Rep. 2018 Mar 21;8:4983. doi: 10.1038/s41598-018-23223-7 (PMC5862879; doi:10.1038/s41598-018-23223-7)

**The Role of Diacylglycerol Acyltransferase** (**DGAT) 1 and 2 in Cardiac Metabolism and Function**

Nathan D. Roe^a, 1^, Michal K. Handzlik^a, 1^, Tao Li^a^, and Rong Tian^a, *^

**SUPPLEMENTARY FIGURE LEGENDS**

**Supplementary Figure 1.** Cardiac-specific DGAT1 inactivation does not affect basal or dobutamine-challenged cardiac function. Heart rate (A) and fractional shortening (B) at baseline and following i.p dobutamine function. * P < 0.05 vs. baseline.

**Supplementary Figure 2.** Cardiac-specific DGAT1 inactivation does not affect body weight change following HFD. Body weight change over time (A) and percent of body weight gain (B) in control and iKO mice following HFD. Data are presented as mean ± SEM (n=9-15). * P < 0.05 vs. corresponding control diet group.

**Supplementary Figure 3.** Inhibition of DGAT2 or coinhibition of DGAT1 and 2 in the heart does not affect cardiac triglyceride content (A) or function [rate pressure product (RPP) B]. ^13^C isotopomer analysis of glutamate revealed increased fatty acid and decreased glucose oxidation in iKO hearts perfused with DGAT2 inhibitor or vehicle. The dotted line indicates the control level (C). Data are presented as mean ± SEM (n=3-7). * P < 0.05 vs. corresponding control group.

**Supplementary Figure 4.** Full length blots from Figure 1B. The left membrane was exposed for 10 seconds and the right one for 20 seconds. The membranes were cut into straps corresponding to molecular weight of DGAT protein (above 50 kDa) and GAPDH (at or below 37 kDa). The order of samples was the same on all blots. Ten samples in total were run on two different gels (6 samples on one gel and 4 on the other). The top DGAT straps (designated as DGAT1; and their corresponding GADPH straps, designated as GAP1) contain 6 samples on top gel and 4 samples on lower gel. The order of the samples was as follows on top and bottom gel of each protein.

TOP GEL: WT, WT, iKO, iKO, cKO, cKO

BOTTOM GEL: WT, WT, iKO, cKO

Supplementary table 1

Supplementary table 2

Figure S1.

Figure S2.

Figure S3.

Fig. S4


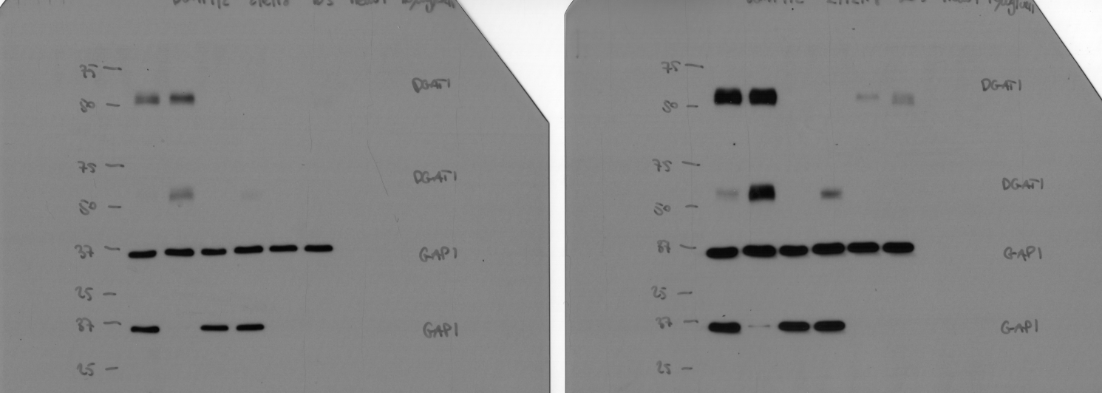

Supplement: Supplementary file 1 — Supplementary Dataset [file 41598_2018_23223_MOESM1_ESM.doc]
